# Bibliographical Notices

**Published:** 1855-08

**Authors:** 


### BIBLIOGRAPHICAL NOTICES.

*A Monograph on Mental Unsoundness.* By FRANCIS WHARTON. Philadelphia: Kay and Brother. 1855.

The analytical table which Mr. Wharton has prefixed to this work is admirably arranged. It shows the reader, at a glance, the manner in which this difficult subject is to be discussed, the relation of each part to the other, of each part to the whole, and greatly enhances the value of the work to the student, and as a book of reference.

The work is divided into two chapters; the first treats of "Mental Unsoundness in its legal relations," in which the law as it now exists in this country is fully laid down under four heads, viz:

I. What degree of unsoundness invalidates a contract or will.

II. What is necessary to be proved in order to deprive a party of the management of his estate.

III. What degree of unsoundness avoids responsibility for crime.

IV. How far intoxication affects responsibility for crime.

When our statutes, or the decisions of our courts, are at variance with the English law, the fact is noticed, and great labor has been given by Mr. Wharton to prove by citation of authorities that his view of the common law is correct.

We fully agree with him as to the great care and delicacy, with which the question of setting aside the will of the aged or partially deranged should be approached. After quoting from De Boismont to the effect that a person should not be deprived of liberty and the right to make a will on account of whimsical actions or words, the result of an erroneous belief, he says:

"Nor should it be forgotten that the effects of such incapacitation would be most cruel to the sufferer himself. Society is prone enough to make eccentricities and weaknesses the subject of contempt, ridicule or insult. The courts should be cautious how, by taking away the power to ensure respect, they thus increase the misfortune of a class into which no man can assure himself he may not fall—which includes almost the whole of those whose lot it is to reach extreme old age—and which already carries a burden sufficiently heavy. If such persons cannot reward by their bounty those by whom they are treated with tenderness, and by whose means their comfort is guarded, they will lose, in most instances, the only means remaining to them of self preservation. The law which thus deprives them of their own means of self support, should tender them in return a refuge by which, by public sanction, they could exact that attention which their own influence no longer enables them to secure. This, however, is certainly not done now, nor could it be done on any intelligible basis, without consigning a large proportion of the most efficient members of society to an asylum. As society at present stands, the only remedy seems to be to throw the same tender guardianship around the feeble minded and the eccentric, as in a passage already cited has been so touchingly invoked by Chancellor Kent for the old."

The question of how Intoxication affects responsibility for crime is one of great importance. Mr. Wharton has handled it very ably. The law, he says, may be summed up as follows:

"1st. Insanity, produced by *delirium tremens*, affects responsibility in the same way, as insanity produced by any other cause.

2d. Insanity, immediately produced by intoxication, does not destroy responsibility, if the patient, when sane and responsible, makes himself intoxicated.

3d. While drunkenness *per se* is no defence to the *fact* of guilt, yet when the question of *intent* or *premeditation* is concerned, it becomes a material item of consideration."

In the second chapter, under the head of "Mental Unsoundness considered psychologically," he treats of the points in which psychology comes in contact with the law of the land. Our limits forbid our discussing as fully as we wish Mr. Wharton's view of this interesting subject.

He states that the experience of medical experts, like that of experts in all other branches of science, is part of the law of the land; it becomes, therefore, of the greatest importance that there should be a full understanding between the legal and medical professions in any case that comes before them, and that they also should act in common.

The following reasons for the consideration and respect which should be paid to the evidence of such experts in questionable cases will be read with interest. Mr. Wharton says:

"It is not to be denied that a lay observer, or an unassisted judge or jury, may be able to distinguish a case of fully developed and clearly manifested insanity; but, aside from the necessity of a knowledge of all the particular relations existing between a given state of disease and a given act, there can be no proper foundation for the infliction of punishment in any case, where the judgment of which it is the execution is not based on the greatest attainable amount of certainty. But this certainty can be no other than that which bears the seal of technical science. Nor will a juryman, if properly tender of his conscience and of public opinion, base his verdict upon other evidence than that of those best able, from long training and close attention, to understand the features of the case. In some cases the difference between a scientific, or technical, opinion, and that of a layman, is not so much in the results attained, as in the guarantee afforded by the superior attainments and more minute expertness of the man of science. The declaration of such a man is insured against the possibility of error to the full extent of the protection of science in its present stage of development. *Pro foro* this degree of certainty is sufficient, because it is the highest attainable; but the same cannot be said of any other."

Monomania is considered under nine heads; we think another should have been added, viz: oinomania, or a morbid taste for liquor, which is sometimes produced by abuse of alchoholic and vinous drinks, and sometimes appears to be congenital, in either case overcoming the will and reason of the person affected, who often deplores his depraved taste, tries to resist, and for a time may, but any unusual excitement, accidentally tasting, or sometimes even the sight or smell of liquor, brings on a desire for alcoholic drinks, against which the will is powerless. Where this constitutional or acquired tendency is known to exist, it appears to us the law should make the same allowance for crime committed in a drunken fit that it now does when crime is committed by a person suffering from an attack of delirium tremens.

The 12th and last section of the 2d chapter is on the treatment of insane criminals. We entirely agree with the author as to the necessity of amending the law as proposed in the following extract, in which also are shown the evils of the present system.

"If the views taken in the preceding sections be sound,—if, in the first place, there are inherent difficulties in the way of making insanity a ground of defence on the trial of a man, who, on this hypothesis is psychologically incapable of either tendering or preparing any such issue; if, in the second place, the doctrine of instinctive or moral mania be allowed in legal theory the sweep which is asserted for it by medical experts, and which in this country at least is conceded to it by the Courts; if, in the third place, it be right that the present system of confinement of insane criminals be remodelled,—then it will become necessary for those to whom the work of legislation is committed to amend the law so as to reserve the question of insanity to be determined by a competent tribunal *after* a conviction of the fact of guilt. For the following undeniable evils result from the present system:

(a.) A tribunal of, at least, but secondary competency is charged with the determination of the most difficult and yet most momentous question to which human observation can be applied.*

(b.) A subject is introduced into the question of guilt or innocence, as to which no fixed judicial rules can be laid down, and which really concerns only the character and the extent of punishment.

(c.) A fearful confusion takes place between the *sane* convict; the *insane* malignant convict, who requires discipline and is, in some degree, morally responsible; the *innocent* insane convict;† and the lunatic, who is in confinement, but is not charged with crime: for all of whom there is in some jurisdictions but *one* common method of discipline provided, viz: that of the penitentiary; in others, but *two*, that of the penitentiary and of the ordinary lunatic asylum. The result of this is acquittals in some cases, when there should be convictions; convictions in other cases when there should be acquittals, and in almost all cases an erroneous system of punishment.

The remedy for these difficulties is one to which we must come sooner or later, and for which the common law has been from the beginning always striving, and yet always losing from almost its very grasp. It is to confine the inquiry before the court and jury to the mere *factum* of the commission of the offence; reserving the question of treatment to be determined by a special commission of experts, to be appointed for the purpose of examining convicts alleged to be insane. The proposition to be put by the court to the jury, under such circumstances, is not, "Was the defendant capable of judging between right and wrong," a proposition which no jury can determine, but, "Did he," as a matter of fact, "commit the specific act charged." For whether he committed it as sane or insane, the result is, if the offence in point of law be indictable, that the safety of society requires that he should be placed in

* Dr. Hood very justly remarks, "All human tribunals are fallible, and how when this plea of insanity is raised, can we unveil the mind of the accused, and determine where responsibility ends and irresponsibility begins? We may appreciate outward and visible signs, but we have no *mentometer*, (if I may be allowed to coin a word) which will indicate the thoughts that may be passing through the mind. In medical jurisprudence the diagnosis between sanity and insanity is, in many cases, infinitely difficult; and it is upon this account that specialists in this branch of our profession so often come into collision with members of the bar, and draw down upon themselves occasionally animadversions from the judges on the bench. There would be no difference of opinion between the two learned professions if we could arrive at any fixed principles by which we could explain the silent operations of the mind; but this, so far as insanity is concerned, is as impossible in law as it is in medicine. We may adjudicate upon the overt act, but the motive which dictated it will very often elude the most searching examination. But this happens continually in sane as well insane life." Suggestions for the future Provision of Criminal Lunatics, by W. C. Hood, M. D. London, 1854.

And we may add to this the testimony of a great poet on a kindred point:

"May it please your Excellency, your thief looks
Exactly like the rest, or rather better;
'Tis only at the bar *or in the dungeon*
That wise men know your felon by his features."

† See as to distinction between these, *ante*, § 232–251.

seclusion for such a period as will promote the joint ends of personal reformation and the preservation of the well-being of the community at large. If he be guilty without the palliation of mental infirmity, certainly the severest penal code—with the single qualification of cases of murder in the first degree,—can ask nothing more than this. If, on the other hand, he was at the time laboring under mental derangement, in no other way can the extent of his responsibility be accurately determined, and the proper degree of discipline adjusted. For this great question of sanity or insanity can really be only determined by those to whose daily and hourly care the convict is committed, and who have thus full opportunity of inquiring into his antecedent as well as his present condition. 'Thus,' to adopt the language of a late very intelligent commentator, 'except as regards the curative course to be adopted, on our view of the case, the subtle line of distinction which there have been so many abortive attempts to draw, between criminal and non-criminal lunatics is of no practical importance, and the unavailing search, unless as a matter of metaphysical speculation, may be abandoned as unnecessary. In either case, the person concerned, whether called a lunatic, a criminal lunatic, or an ordinary criminal, should be so placed as to put it out of his power to inflict further injury, and to afford the most likely means for his cure.' And thus, also, not only will the sanctions of human life and property be protected from the recurrence of those monstrous acquittals, by which, under the plea of insanity, the most dangerous criminals are suffered to run at large, but the interests of humanity will be subserved by a proper discipline, as well as a just classification, of those whose accountability is diminished or destroyed."

Mr. Wharton's work cannot fail to interest the general as well as the professional reader. His style is clear and forcible, the matter treated of affects us all more or less nearly, and is throughout illustrated by cases of thrilling interest. We warmly recommend it to our readers.

---

*A Practical Treatise on the Diseases, Injuries, and Malformations of the Urinary Bladder, the Prostrate Gland, and the Urethra.* By S. D. Gross, M.D., Professor of Surgery in the University of Louisville, &c. Second Edition, Revised and much Enlarged. Philadelphia: Blanchard & Lea. 1855.

The very flattering reception with which the first edition of the above work was received by the profession, both at home and abroad, renders it unnecessary for us to say anything of its merits. The present edition has been enriched by upwards of two hundred pages of additional matter, which are dispersed throughout in the form of chapters, sections and paragraphs, imparting to it somewhat of the aspect of a new treatise. It is now quite a large book, comprising more than nine hundred pages ; this, however, is the less to be regretted, as the author has been enabled by its increased size to enter much more satisfactorily into the detail of the diseases and injuries of which it treats than otherwise he could have done. As it now stands, we consider it the most valuable and thoroughly complete treatise on diseases of the urinary organs in the English language. It is in every respect a work to be proud of. The vast amount of information contained in its pages, its clear and simple style, and the sound judgment which presides in all its teachings, leave nothing whatever to be desired by the reader.

The work is gotten up in excellent style by the enterprising publishers, and is embellished by one hundred and eighty four illustrations.

---

*Mineral and Thermal Springs of the United States and Canada.* By JOHN BELL, M.D., author of "Baths and Mineral Waters ;" "Baths and the Water Regimen ;" "Lectures on the Practice of Physic," &c. Philadelphia: Parry & M'Millan. 1855.

We need only draw the attention of our readers to the above useful and interesting little work to secure it a favorable reception. Dr. Bell, it is well known, has devoted much time and reflection to the subject of baths and mineral waters, and their effects upon the human system. The present volume, though but a small one, fully sustains the character for judgment and knowledge of his former writings. A great deal of necessary and agreeable information is contained in its pages, and it is equally well adapted to the comprehension of the general and professional reader.

"The want of a manual in which travellers for curiosity and pleasure, and invalids in quest of health, might learn where to go, how to go, and what to find in relation to the Mineral and Thermal Springs of our country has been generally felt. Physicians, too, have wished for a work to which they could refer for information respecting the physical and chemical properties and medicinal virtues of the several springs. There are, indeed, some published accounts of particular springs, and even groups of springs, as, for example, of those of New York and of

Virginia, of which free use has been made in the following pages. But, with the exception of a work by the author issued twenty-five years ago, no attempt has hitherto been made to collect and arrange methodically the numerous separate and scattered histories and descriptions of the different mineral and thermal springs of the United States, as has been done for those of Great Britain and Ireland, of France, of Germany, and, in a more restricted manner, of Italy."

"It contains notices, more or less full, of one hundred and thirty springs and groups of springs belonging to the United States. If account were taken of each separate spring of the several groups, which is marked by distinctive properties, the number would exceed two hundred. Of those described, there are, as will be seen in the tabular view presented in the appendix, about thirty of the thermal class, a great majority of which must be quite new to most readers."

We advise travellers to our various watering places to provide themselves with a copy of the above volume, as there are very few of them who will not derive advantage from its perusal. The directions for drinking the different waters, and for bathing, are full and judicious, and may be understood by all. The book has a copious index.

---

*A Pocket Formulary and Physician's Manual, embracing the Art of Combining and Prescribing Medicines to the Best Advantage; with many Valuable Recipes, Tables, &c.* By THOMAS S. POWELL, M.D., of Sparta, Georgia. Savannah: W. Thorne Williams. 1855.

This little work is one of the best arranged of its class, and will, we are confident, be found especially adapted to the wants of the American practitioner. The author is one of the most respectable and experienced physicians of our Southern States, and is thoroughly qualified for the task of preparing a "Pocket Formulary," adapted to the occasions of the profession of our country.

---

*Medical Lexicon, or Modern Terminology; being a Complete Vocabulary of Definitions, including all the Technical Terms employed by Writers and Teachers of Medical Science at the present day. Third Edition.* By D. MEREDITH REESE, M.D., &c. New York: S. S. & W. Wood. 1855.

That this useful little work has supplied a want of students, is shown by its having reached a third edition. As a vocabulary of definitions, of which the briefest possible is given in every instance, it is well fitted for the purpose it is intended for—a help to students and young practitioners.

